# Supplementary material for: Genetic comparisons of interleukin-17 reveal a framework for complex signaling evolution
Source: bioRxiv. 2026 Apr 14:2026.04.13.718218. Preprint. [Version 1] doi: 10.64898/2026.04.13.718218 (PMC13105070; doi:10.64898/2026.04.13.718218)
Supplement: Supplement 3 [file media-3.zip › Supplemental files for paper/ERC/IL17xIL17_comparisons/IL17REL_IL17F.pdf]

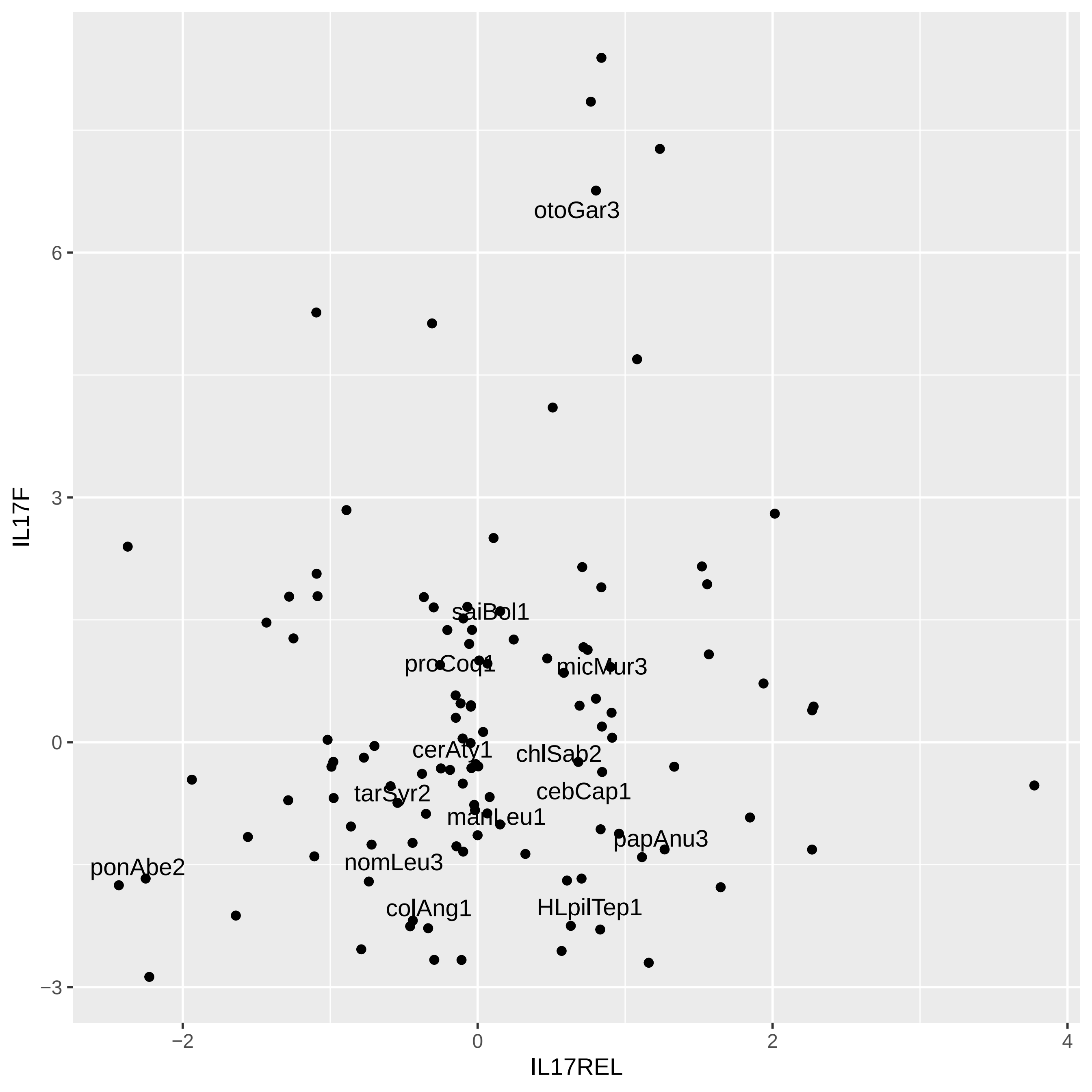
otoGar3
saiBol1
proCoq1
micMur3
cerAty1
chlSab2
tarSyr2
cebCap1
manLeu1
papAnu3
ponAbe2
nomLeu3
colAng1
HLpilTep1
6
3
0
−3
−2
0
2
4
IL17F
IL17REL
